# The Characterization of a Piston Displacement-Type Flowmeter Calibration Facility and the Calibration and Use of Pulsed Output Type Flowmeters

**DOI:** 10.6028/jres.097.023

**Published:** 1992

**Authors:** G. E. Mattingly

**Affiliations:** National Institute of Standards and Technology, Gaithersburg, MD 20899

**Keywords:** flow measurement, fluid meter characterization, meter performance, output flowmeter, turbine flowmeter, volumetric calibrator

## Abstract

Critical measurement performance of fluid flowmeters requires proper and quantified verification data. These data should be generated using calibration and traceability techniques established for these verification purposes. In these calibration techniques, the calibration facility should be well-characterized and its components and performance properly traced to pertinent higher standards. The use of this calibrator to calibrate flowmeters should be appropriately established and the manner in which the calibrated flowmeter is used should be specified in accord with the conditions of the calibration.

These three steps: 1) characterizing the calibration facility itself, 2) using the characterized facility to calibrate a flowmeter, and 3) using the calibrated flowmeter to make a measurement are described and the pertinent equations are given for an encoded-stroke, piston displacement-type calibrator and a pulsed output flowmeter. It is concluded that, given these equations and proper instrumentation of this type of calibrator, very high levels of performance can be attained and, in turn, these can be used to achieve high fluid flow rate measurement accuracy with pulsed output flowmeters.

## 1. Introduction

Fluid flowmeter calibration methods use a variety of techniques; they include wide ranges of operational parameters; they cover wide ranges of precision and accuracy, [1–3]. Increasingly, the diverse improvements being sought for flowmeters are producing corresponding improvements in the characteristics of flowmeter calibration systems. Of these systems, the piston displacement-type calibrator offers advantages such as compactness, mobility, efficient change of fluid, and prospects for state-of-the-art performance characteristics.

To characterize the performance of a piston displacement calibrator, which produces a pulsed output signal that is proportional to the volumetric flowrate, the objective is, generally, to determine the “pulses per volume displacement” ratio (or its reciprocal) where the pulse output is assumed to come from a source such as a linear encoder. This pulse output is also assumed to be proportional to the piston displacement. High accuracy calibrator performance requires examination of these assumptions.

A number of techniques can be used to determine volumetric displacement. It is assumed in what follows that the displaced fluid is a liquid, but the principles apply to gases or mixtures of gases and liquids as well. It is also assumed that both temperature and pressure effects on all the components of the piston displacement system, the cylinder, encoder, and fluid should be considered in order that high accuracy performance can be achieved.

Depending upon the desired uncertainty level for the performance of the calibrator, one or more of the pressure and temperature effects on the components of the system may be negligible. When this is so, it may be permissible to disregard such effects to simplify data processing or to reduce the size of the controlling software for the system. Alternatively, and more preferably, all effects can be included in computer software. In this way, the terms which are negligible will not influence the results when higher levels of uncertainty, i.e., less precise performance can be tolerated or is desirable from benefits vs. costs perspectives. More importantly, where high accuracy is required, more of the figures available via the software capabilities can be accepted as significant.

**Fig. 1:**
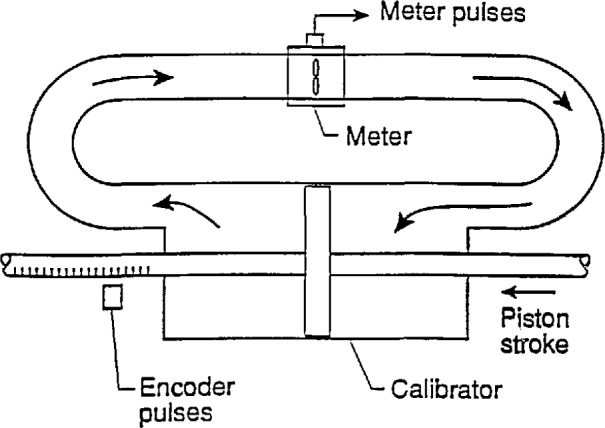
Sketch of cncodcd-strokc calibrator in operation.

The volumetric-type calibrator system, using encoded piston displacement as both the flow source and as the flow determination scheme, is sketched in Fig. 1. The piston motion produces and measures a fluid volumetric flowrate that is proportional to the encoder frequency. The piston in the cylinder has a seal that is assumed to seal perfectly for all piston velocities. The corresponding fluid flowrate through the meter and the meter output frequency enable a calibration of the flowmeter. Using these elements, three steps are considered.

The first step is determining the calibrator factor which is an “encoder pulses per volume displaced” ratio (or its reciprocal) at the defined set of reference conditions. These results can be obtained experimentally in a number of ways: (1) by physical measurement, (2) by the so-called “draw” technique, or (3) by using a transfer standard such as a single or, preferably, a tandem arrangement of calibrated turbine flowmeters.

The second step is the use of the characterized calibrator to calibrate a pulsed-output flowmeter such as a turbine meter or other device where the meter factor is a “pulses per volume” quantity or its reciprocal. This process is done with one or more selected fluids and at specified conditions. In what follows turbine flowmeters will be considered. Conventionally, turbine meter results are produced in the form of a meter factor which has units of pulses per volume or volume per pulses (referenced to specified conditions). This meter factor is determined over the desired ranges of fluid conditions and flowrate expressed in terms of a ratio of inertial-to-viscous effects such as a Reynolds number, or equivalent parameter.

The third step is the use of the characterized turbine flowmeter to calculate a fluid flowrate under actual conditions of use. The results can be produced with respect to specified reference conditions or to the actual conditions, depending upon the needs of the meter operator.

The purpose of this paper is to describe these three steps and give the pertinent relationships that pertain to each procedure. The resulting equations are intended to be used in the software packages used with these types of calibrators and metering units. In this way, it is expected that the measurement performance of both the calibrators and the metering units can be maximized.

## 2. Calibrator Characterization

### 2.1 Geometrical Determination at Reference Conditions

**Fig. 2:**
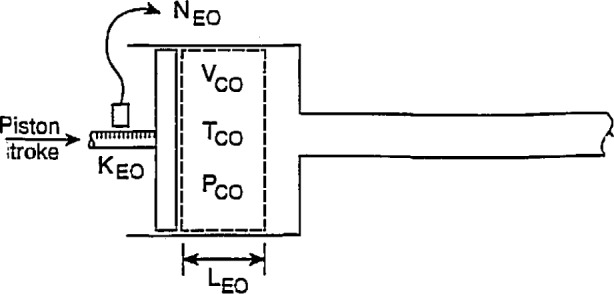
Encoded-stroke calibrator configuration at reference conditions.

To perform the required measurements at reference conditions and then calculate the calibrator factor in units of pulses per fluid volume displaced, the system analyzed is that sketched in Fig. 2. The assumption of reference conditions is a conceptual situation that is impractical to achieve precisely but is done solely for convenience, as will be clear in what follows. For the conditions selected to be the reference conditions which are denoted by the “0” subscript, a specific piston stroke produces a displacement volume, *V*_C0_, and the encoder produces the corresponding number of pulses, *N*_E0_. A list of symbols is given in the Glossary. The reference conditions of calibrator temperature and the pressure in the calibrator are *T*_C0_ and P_C0_, respectively. These properties are assumed to be constant and steady in the calibrator volume. These conditions should be monitored, quantified, and assessed with respect to the performance level of the calibrator. Furthermore, the fluid inside and outside the calibrator is assumed to have the same temperature as the cylinder of the calibrator and no heat is being transferred to or from the calibrator. In all that follows, the reference conditions of *T*_0_ and *P*_0_ will be assumed to be the same for all components.

The specified piston stroke, *L*_E0_, produces the pulse total, *N*_E0_, where

$$N_{E0}=L_{E0}K_{E0},$$ (1)

and *K*_E0_ is the encoder constant in pulses per length at the reference temperature condition, *T_0_.* The calibration constant can be written

$$K_{C0}=\frac{N_{E0}}{V_{C0}}=\frac{L_{E0}K_{E0}}{{\bar{A}}_{C0}L_{E0}}=\frac{K_{E0}}{{\bar{A}}_{C0}},$$ (2)

in units of pulses per volume, where, at the reference conditions, *Ā*_C0_ is the averaged cylinder cross-sectional area over the piston stroke, *L*_E0_. This calibrator constant, *K*_C0_ is assumed to be constant over the operating range of the calibrator.

For the calibrator configuration shown in Fig. 1, the area *Ā*_C0_ is an annular one between the cylinder of the calibrator and the rod or tube attached to the piston. The precision with which *K*_C0_ is determined can be written using root-sum-square combinations of component precisions:

$$\begin{array}{l} \frac{\Delta K_{C0}}{K_{C0}}<{\left[{\left(\frac{\Delta N_{E0}}{N_{E0}}\right)}^{2}+{\left(\frac{\Delta V_{C0}}{V_{C0}}\right)}^{2}\right]}^{1/2}\\ ={\left[{\left(\frac{\Delta K_{E0}}{K_{E0}}\right)}^{2}+{\left(\frac{\Delta {\bar{A}}_{C0}}{{\bar{A}}_{C0}}\right)}^{2}\right]}^{1/2}, \end{array}$$ (3)

where the numerators of the respective terms refer to the maximum errors of each of the component measurements. Equation (3) indicates that high levels of precision in *K*_C0_ can be attained when large pulse sums, *N*_E0_, and large displaced volumes, *V*_C0_, are used. Correspondingly, these precision levels can be achieved with accurate and sensitive linear encoders and accurately measured and large cross-sectional areas.

### 2.2 Geometrical Determination at Non-Reference Conditions

**Fig. 3:**
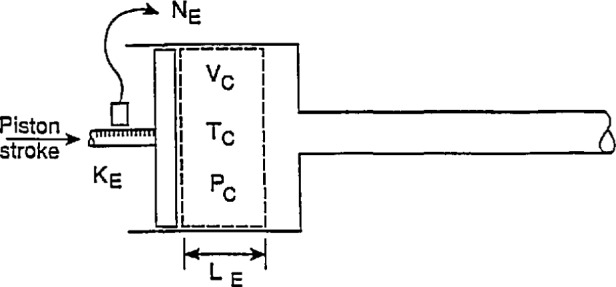
Encoded-stroke calibrator configuration at non-reference conditions.

To perform the required measurements at non-reference temperature and pressure conditions, it is assumed that these conditions are constant and steady as shown in Fig. 3. For a specified piston stroke, *L*_E_, the corresponding encoder pulse total, *N*_E_, is

$$N_{E}=N_{E}K_{E},$$ (4)

where the encoder constant, *K*_E_, is assumed to depend only on temperature according to

$$K_{E}=K_{E0}[1-\alpha_{E}\left(T_{E}-T_{E0}\right),$$ (5)

where *α*_E_ is the pertinent linear expansion coefficient for the encoder and *T*_E_ and *T*_E0_ are, respectively, the encoder temperatures at non-reference and reference conditions.

The cross-sectional area change of the calibrator cylinder that is produced by temperature, *T*_C_, and internal fluid pressure, *P*_C_, in excess of the reference values is analyzed in Appendix A. In what follows, it is assumed that the annular area contained between the calibrator cylinder and the rod or tube connected to the piston is changed with temperature in the usual way and the pressure effect is considered to enlarge only the cylinder of the calibrator. The pressure effect on the tube or rod is taken to be negligible. The cross-sectional area of the cylinder of the calibrator averaged over the stroke length is given, to first order in temperature and pressure separately, by

$${\bar{A}}_{C}={\bar{A}}_{C0}\left[1+2\alpha_{C}\left(T_{C}-T_{0}\right)\right]\left[1+\frac{\left(P_{C}-P_{0}\right)D_{C0}}{t_{C0}E_{C}}\right],$$ (6)

where *α*_C_ is the linear expansion coefficient for the material of the calibrator cylinder, *D*_C0_ and *t*_C0_ are, respectively, the inside diameter and wall thickness of the calibrator cylinder at reference conditions and *E*_C_ is the modulus of elasticity of the material of the calibrator cylinder. In Eq. (6) the compressive effects of the pressure in the cylinder on the rod attached to the piston are assumed negligible.

If it is assumed that the linear expansion coefficient of the cylinder material is 2 × 10^−5^ °C^−1^, a systematic temperature difference of only 2.5 °C would produce a corresponding systematic error of±0.01% in the calibrator area. Similarly, if it is assumed that the modulus of elasticity for the calibrator material is 2×10^11^ Pa (2×l0^6^atm) and if the calibrator has a diameter to thickness ratio of 20, a systematic pressure error of only 1 MPa (10 atm) will produce a systematic error of±0.01% in the calibrator area. Such calibrator area errors will propagate through all of the successive relationships for calibrating and using meters, and these relationships will be further altered by additional temperature and pressure effects.

The calibrator constant, *K*_C_, at non-reference conditions in units of pulses per volume can be written

$$K_{C}\frac{N_{E}}{V_{C}},$$ (7)

since *V*_C_ is the displacement at non-reference conditions. Therefore

$$K_{C}=\frac{K_{E}}{{\bar{A}}_{C}}.$$ (8)

Combining Eq. (8) with Eqs. (2), (5), and (6) produces

$$K_{C}=\frac{K_{C0}\left[1-\alpha_{E}\left(T_{E}-T_{0}\right)\right]}{\left[1+2\alpha_{C}(T_{C}-T_{0}\right]\left[1+\frac{\left(P_{C}-P_{0}\right)D_{C0}}{t_{C0}E_{C}}\right]},$$ (9)

or, to first order approximation:

$$K_{C0}=K_{C}\frac{\left[1+\alpha_{E}\left(T_{E}-T_{0}\right)\right]}{\left[1-2\alpha_{C}(T_{C}-T_{0}\right]\left[1-\frac{\left(P_{C}-P_{0}\right)D_{C0}}{t_{C0}E_{C}}\right]}.$$ (10)

For temperatures and pressures higher than reference conditions the calibrator constant, *K*_C_, is less than the value at reference conditions. In both of the above geometrical methods for obtaining calibrator constants, fluid properties are not involved.

The value for *K*_C0_ given by Eqs. (2) or (10) should be put into the software that operates the displacement calibrator, together with pertinent material constants and component dimensions. Then the computation for the calibrator constant *K*_C_ at non-reference conditions can be done using appropriate measurements for encoder temperature, in addition to the pressure and temperature in the calibrator, as shown in Eq. (9). These values of *K*_C0_ or *K*_C_ will be used for the accurate calibrations of flowmeters.

### 2.3 Determination of Calibrator Constant by the Draw Technique at Reference Conditions

**Fig. 4:**
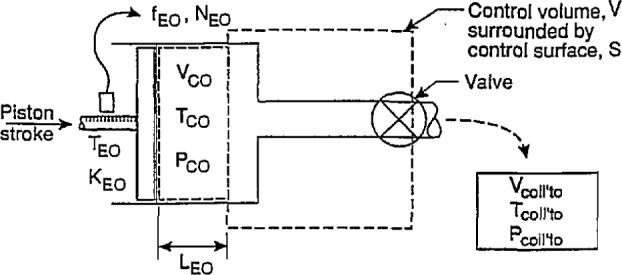
Experimental arrangement for the draw technique at reference conditions.

The configuration for the draw technique is shown in Fig. 4, where the piston stroke displaces an amount of fluid through a valve into the collection tank; the corresponding encoder pulses total N_EO_. All temperatures and pressures are the reference values. In all that follows, it is assumed that the seal of the piston in the calibrator seals perfectly and is sufficiently pliant to continue to seal perfectly when changes in temperature and internal pressure occur and change the diameter of the cylinder of the calibrator. Applying conservation of mass principles to the constant volume shown in figure 4, we obtain:

$$\frac{\partial }{\partial t}\int_{V}\rho dV-\int_{s}\rho \upsilon \cdot n\;dS=0,$$ (11)

where *ρ* is the fluid density, *V* is the control volume surrounded by the control surface, *S*,***υ*** is the fluid velocity vector, and ***n*** is the unit vector normal to *S* with positive direction pointing to the interior of *V.* When

$$\frac{\partial }{\partial t}\int_{V}\rho dV=0,$$ (12)

there is no change of the mass within the control volume in time. This means the effects of fluid friction or of heat transfer are negligible or, taken in total, do not alter in time the mass contained within the control volume. Under such conditions, the inlet and outlet mass fluxes through the control surface, *S* are the same, that is

$$\rho_{C0}\;{\dot{V}}_{C0}=\rho_{{\mathrm{COLL}}^{’}T0\;}{\dot{V}}_{{\mathrm{COLL}}^{’}T0},$$ (13)

where *ρ*_C0_ and *ρ*_COLL’T0_ are the fluid densities, respectively, in the calibrator and collection tank at the same reference conditions and therefore are equal. The quantities ${\dot{V}}_{C0}$ and ${\dot{V}}_{{\mathrm{COLL}}^{’}T0}$ are the volumetric flowrates out of the calibrator and into the collection tank, respectively.

Therefore, since the times of displacement and collection are assumed to be the same,

$$V_{C0}=V_{{\mathrm{COLL}}^{’}T0},$$ (14)

and the calibrator constant, in units of pulses per displacement volume can be written

$$K_{C0}=N_{E0}/V_{{\mathrm{COLL}}^{’}T0},$$ (15)

where

$$V_{{\mathrm{COLL}}^{’}T0}={\bar{A}}_{C0}L_{E0}.$$ (16)

Therefore, using Eq. (13)–(16), we obtain

$$K_{C0}=K_{E0}/{\bar{A}}_{C0},$$ (17)

in agreement with Eq. (2). Accordingly, results obtained via this procedure should duplicate those obtained via the geometrical measurement techniques.

It is generally assumed that the calibrator constant *K*_C0_ is independent of the piston speed or the fluid properties. If this assumption is not valid then it is necessary to characterize the dependence. This should be done in a manner analogous to the procedures used to characterize turbine meters as given below.

It should be noted that, for the control volume shown in Fig. 4, if

$$\frac{\partial }{\partial t}\int_{V}\rho dV\neq 0,$$ (18)

then the mass flux displaced in the cylinder of the calibrator is not equal to that delivered to the collection tank. Furthermore, if the fluid density in the control volume decreases because of a temperature rise then the mass flux to the collection tank would be more than that displaced in the calibrator cylinder. As will be seen below, this produces a turbine-type meter calibration result in pulses per volume units that is larger than it should be. Conversely, if the fluid density in the control volume increases, then the mass flux delivered to the collection vessel is less than that displaced in the calibrator cylinder. This produces a low calibration result for a turbine meter constant in pulses per volume units.

### 2.4 Determination of Calibrator Constant by the Draw Technique at Non-Reference Conditions

**Fig. 5:**
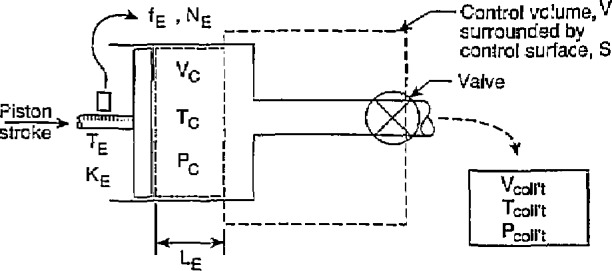
Experimental arrangement for the draw technique at non-reference conditions.

The configuration for the draw technique in non-reference conditions is shown in Fig. 5. As before, the piston stroke displaces a volume of fluid in the cylinder and the corresponding encoder pulses total *N*_E_. The temperature and pressure of the fluid in the cylinder are *T*_C_ and *P*_C_, respectively. Applying conservation of mass principles to the control volume in Fig. 5, for the condition where there is no change with time of the mass within the control volume, Eq. (11) and (12) indicate that

$$\rho_{C}{\dot{V}}_{C}=\rho_{{\mathrm{COLL}}^{’}T}{\dot{V}}_{{\mathrm{COLL}}^{’}T},$$ (19)

or when the displacement and collected volumes are simultaneous

$$\rho_{C}V_{C}=\rho_{{\mathrm{COLL}}^{’}T}V_{{\mathrm{COLL}}^{’}T},$$ (20)

where *ρ*_C_ and *ρ*_COLL’T_ are, respectively, the density of the fluid displaced within the cylinder of the calibrator and flowing from the valve and into the collection tank. The volume *V*_COLL’T_ can be determined using volumetric or gravimetric techniques, or both. It is noted that constant conditions of temperature and pressure need to be maintained in the calibrator and in the collection vessel for accurate results.

With the volume *V*_COLL’T_ determined, *V*_C_ can be computed via

$$V_{C}=\left(\rho_{{\mathrm{COLL}}^{’}T}/\rho_{C}\right)V_{{\mathrm{COLL}}^{’}T},$$ (21)

where, according to the definitions of fluid thermal expansion effects and compressibility, to first order in temperature and pressure, separately:

$$\begin{array}{l} \frac{\rho_{{\mathrm{COLL}}^{’}T}}{\rho_{C}}=\\ \left[1-3\alpha_{F}\left(T_{{\mathrm{COLL}}^{’}T}-T_{C}\right)\right][1+\frac{\left(P_{{\mathrm{COLL}}^{’}T}-P_{C}\right)}{E_{F}}], \end{array}$$ (22)

where *α*_F_ is the linear expansion coefficient for the fluid, the pressures *P*_COLL’T_ and *P*_C_ are, respectively, those for the fluid passing from the valve into the collection tank and in the cylinder of the calibrator, and *E*_F_ is the modulus of elasticity of the fluid. It is noted that both α_F_ and *E*_F_ are dependent upon temperature and pressure for the specific fluid, but the values used here and in what follows are assumed to be averages taken over the appropriate ranges of temperature and pressure. It is also noted here that the reciprocal of the modulus of elasticity of the fluid is also the compressibility of the fluid.

In Eq. (22), it is noted that temperature and pressure effects have opposite signs in producing fluid density changes. However, when the temperature differences between the collection vessel and the calibrator are large and where the pressure in the calibrator is much larger than that in the collection vessel, the effects on the fluid can be significant.

If it is assumed that the linear expansion coefficient of a hydrocarbon liquid is 3 × 10^−4^ °C^−1^, a systematic temperature error of only 1 °C will produce a systematic error of 0.1% due to temperature in the determination of the calibrator volume by this draw method. Similarly, if it is assumed that the fluid’s modulus of elasticity is 2 × 10^9^ Pa (2 × 10^4^ atm), a systematic pressure error of only 2 MPa (20 atm) will produce a systematic error of ±0.1% in the calibrator volume. As mentioned above, such errors will propagate through all of the relationships for calibrating and using meters and these errors will be further altered by additional temperature and pressure corrections.

Furthermore, using Eq. (7) we can write

$$K_{C}=\frac{N_{E}}{\left(\frac{\rho_{{\mathrm{COLL}}^{’}T}}{\rho_{C}}\right)V_{{\mathrm{COLL}}^{’}T}},$$ (23)

then, from Eq. (22)

$$\begin{array}{l} K_{C}=\frac{N_{E}}{V_{{\mathrm{COLL}}^{’}T}}\left[1+3\alpha_{F}\left(T_{{\mathrm{COLL}}^{’}T}-T_{C}\right)\right]\\ \times \left[1-\frac{\left(P_{{\mathrm{COLL}}^{’}T}-P_{C}\right)}{E_{F}}\right]. \end{array}$$ (24)

This result is then related to reference conditions via Eq. (10) to give

$$\begin{array}{l} K_{C0}=\\ \frac{N_{E}\left[1+3\alpha_{F}\left(T_{{\mathrm{COLL}}^{’}T}-T_{C}\right)\right]\left[1-\frac{\left(P_{{\mathrm{COLL}}^{’}T}-P_{C}\right)}{E_{F}}\right]\left[1+\alpha_{E}\left(T_{E}-T_{0}\right)\right]}{V_{{\mathrm{COLL}}^{’}T}\left[1-2\alpha_{C}\left(T_{C}-T_{0}\right)\right]\left[1-\frac{\left(P_{C}-P_{0}\right)D_{C0}}{T_{C0}E_{C}}\right].} \end{array}$$ (25)

It is noted that the cross-sectional area, *Ā*_C_ can be determined via Eqs. (8) and (23) to be

$${\bar{A}}_{C}=\frac{K_{E}}{N_{E}}\left(\frac{\rho_{{\mathrm{COLL}}^{’}T}}{\rho_{C}}\right)V_{{\mathrm{COLL}}^{’}T}.$$ (26)

Using Eqs. (5), (6) and (22), this can be written in terms of directly measured quantities and related to reference conditions via

$$\begin{array}{l} {\bar{A}}_{C0}=\\ \frac{K_{E0}V_{{\mathrm{COLL}}^{’}T}\left[1-\alpha_{E}\left(T_{E}-T_{0}\right)\right]\left[1-2\alpha_{C}\left(T_{C}-T_{0}\right)\right]\left[1-\frac{\left(P_{C}-P_{0}\right)D_{C0}}{t_{C0}E_{C}}\right]}{N_{E}\left[1+3\alpha_{F}\left(T_{{\mathrm{COLL}}^{’}T}-T_{C}\right)\right]\left[1-\frac{\left(P_{{\mathrm{COLL}}^{’}T}-P_{C}\right)}{E_{F}}\right].} \end{array}$$ (27)

If *K*_Ć_ is defined as

$$K_{Ć}=N_{E}/V_{{\mathrm{COLL}}^{’}T},$$ (28)

then

$$\begin{array}{l} K_{C0}=K_{Ć}\times \\ \frac{\left[1+2\alpha_{C}\left(T_{C}-T_{0}\right)\right]\left[1+\frac{\left(P_{C}-P_{0}\right)D_{C0}}{t_{C0}E_{C}}\right]}{\left[1-3\alpha_{F}\left(T_{{\mathrm{COLL}}^{’}T}-T_{C}\right)\right]\left[1+\frac{\left(P_{{\mathrm{COLL}}^{’}T}-P_{C}\right)}{E_{F}}\right]\left[1-\alpha_{E}\left(T_{E}-T_{0}\right)\right]} \end{array}$$ (29)

or, to first order

$$\begin{array}{l} K_{Ć}=K_{C0}\times \\ \frac{\left[1-2\alpha_{C}\left(T_{C}-T_{0}\right)\right]\left[1-\frac{\left(P_{C}-P_{0}\right)D_{C0}}{t_{C0}E_{C}}\right]}{\left[1+3\alpha_{F}\left(T_{{\mathrm{COLL}}^{’}T}-T_{C}\right)\right]\left[1-\frac{\left(P_{{\mathrm{COLL}}^{’}T}-P_{C}\right)}{E_{F}}\right]\left[1+\alpha_{E}\left(T_{E}-T_{0}\right)\right]}. \end{array}$$ (30)

When temperatures exceed those of the reference conditions and pressures are those for the reference conditions:

$$K_{Ć}<K_{C0}.$$ (31)

Thus, the observation made above is repeated here, namely, that when pressure effects can be neglected and when temperatures are above the reference conditions, the calibrator delivers, for the same encoder output pulses, more fluid volume than would occur under reference conditions.

It is also noted that Eqs. (23)–(27) can be combined to give:

$$K_{C}=K_{C0}\frac{\left[1-\alpha_{E}\left(T_{E}-T_{0}\right)\right]}{\left[1+2\alpha_{C}\left(T_{C}-T_{0}\right)\right]\left[1+\frac{\left(P_{C}-P_{0}\right)D_{C0}}{t_{C0}E_{C}}\right]},$$ (32)

which is the same as Eq. (9).

It is noted that, in these two draw procedures, the decision to collect a sufficiently large number of encoder pulses should precede the operation. This number should be selected according to the desired precision for the calibrator constant, see Eq. (3). In the reference conditions

$$N_{E0}=K_{E0}L_{E0},$$ (33)

and in the non-reference conditions

$$N_{E}=K_{E}L_{E},$$ (34)

but, to first order in temperature,

$$K_{E}=K_{E0}\left[1-\alpha_{E}\left(T_{E}-T_{0}\right)\right],$$ (35)

and

$$L_{E}=L_{E0}\left[1+\alpha_{E}\left(T_{E}-T_{0}\right)\right].$$ (36)

Combining Eqs. (33)–(36) therefore yields:

$$N_{E}=K_{E}L_{E}=K_{E0}L_{E0}=N_{E0}.$$ (37)

This indicates that the precision criterion specified for the calibrator should be achieved via the number of pulses selected and the choice is not dependent upon whether reference or non-reference conditions prevail.

As stated above following equation (17), the calibrator constants determined via the draw procedure are generally assumed to be independent of piston speed and fluid properties. Where this is not valid, efforts should be made to achieve this assumption, i.e., improving the piston seals or the calibrator should be characterized using techniques analogous to those for turbine meters as will be described below.

It should also be noted that in the above described draw procedures, the valve and pulse counting techniques must not introduce spurious effects. Spurious counting effects may result from the fluid dynamics in the valve as the flow is started and stopped in conjunction with starting and stopping the pulse count. If such an effect is present then it should be eliminated or proper account made for it so that the appropriate fluid volume is associated with the pulse total. This volume can be determined using the valve compensation techniques that are conventionally applied to diverter systems, see [4].

To use the now-characterized calibrator, it is required that appropriate instrumentation be properly installed both to assure that Eq. (12) is satisfied and to measure the quantities involved in Eqs. (9) or (10) or (32) for the calibrator and equations (28) or (29) or (30) for dispensing precise volumes of fluid at specified conditions of temperature and pressure. Appropriate values are needed for the material constants—the thermal expansion coefficients *α*_E_, *α*_F_ and *α*_C_, the modulus of elasticity of the material of the calibrator cylinder, *E*_C_, and that of the fluid, *E*_F_, and the pertinent dimensions of the cylinder. Once the appropriate value for the constant *K*_C0_ has been installed in the calibrator software or the working procedures for the calibrator, the next step is to use the calibrator to calibrate a flowmeter.

## 3. Calibrator Use in Calibrating a Turbine Type Flowmeter

### 3.1 Reference Conditions

**Fig. 6:**
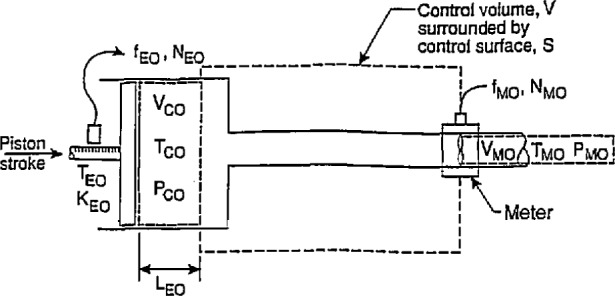
Arrangement for using characterized calibrator to calibrate a turbine-type flowmeter at reference conditions.

To calibrate a turbine-type flowmeter at reference temperature and pressure conditions using the calibrator characterized as described above, the arrangement is sketched in Fig. 6. The temperature and pressure are the reference conditions denoted by *T*_0_ and *P*_0_. As stated above, *T*_0_=*T*_M0_=*T*_C0_ and *P*_0_*=P*_M0_*=P*_C0_. Applying again the conservation of mass principles,. and assuming that there is no change of mass within the control volume with time, Eqs. (11) and (12) indicate that

$$\rho_{C0}{\dot{V}}_{C0}=\rho_{M0}{\dot{V}}_{M0},$$ (38)

where *ρ*_C0_ and *ρ*_M0_ are the fluid densities in the calibrator and meter, respectively, at the reference conditions. The quantities ${\dot{V}}_{C0}$ and ${\dot{V}}_{M0}$ are the volumetric flowrates, respectively, in the calibrator and through the meter at the reference conditions.

Since reference conditions in cylinder and meter are assumed the same, the densities *ρ*_C0_ and *ρ*_M0_ are equal and therefore:

$${\dot{V}}_{C0}={\dot{V}}_{M0}.$$ (39)

Since the pertinent time intervals are assumed the same:

$$K_{C0}=\frac{K_{E0}}{{\bar{A}}_{C0}}=\frac{N_{E0}}{{\bar{A}}_{C0}L_{E0}}=\frac{f_{E0}}{{\dot{V}}_{C0}},$$ (40)

where *f*_E0_ is the encoder frequency, and

$$K_{M0}=\frac{N_{M0}}{V_{M0}}=\frac{f_{M0}}{{\dot{V}}_{M0}},$$ (41)

where *f*_M0_ is the meter frequency.

In Eqs. (40) and (41), the pulse counts *N*_E0_ and *N*_M0_ are to correspond to the respective volumes *V*_C0_ and *V*_M0_ which are assumed to be the same, or appropriate corrections are made to compensate for any differences between these volumes. Combining Eqs. (40) and (41) gives

$$K_{M0}=\frac{f_{M0}}{f_{E0}}\frac{K_{E0}}{{\bar{A}}_{C0}}=\frac{N_{M0}}{N_{E0}}\frac{K_{E0}}{{\bar{A}}_{C0}}=\frac{N_{M0}}{N_{E0}}•K_{C0}=\frac{f_{M0}}{f_{E0}}•K_{C0},$$ (42)

where *K*_M0_has units of pulses per volume at the reference conditions. The quantity *K*_E0_ is obtained from manufacturer’s specifications, testing or Eq. (1).

Conventional non-dimensionalization procedures can be applied to the flowmeter characteristics to produce a set of parameters which interrelate the significant inertial, viscous, and oscillatory effects that constitute the performance of the meter in the calibration conditions, see [6,7]. In this way, the performance of the device can be predicted for other fluid and flow conditions where this set of parameters are the pertinent ones to describe the meter’s performance. Of course, when other, different effects such as fluid compressibility or gravitational influences become significant, it should be expected that the initial parameterization needs to be modified to include such effects to obtain satisfactory description of meter performance, see [8,9].

By normalizing the meter factor, *K*_M0_ and the fluid flowrate using the meter diameter, *D*_M0_ and the mean flow velocity ${\dot{V}}_{M0}/A_{M0}=U_{M0}$ and the fluid kinematic viscosity *υ*_0_, we can obtain, for example, the Strouhal number

$$St=\frac{f_{M0}D_{M0}}{U_{M0}}=C_{1}K_{M0}D_{M0}^{3}\propto K_{M0}D_{M0}^{3},$$ (43)

where *C*_1_=π/4. The Strouhal number is the ratio of characteristic meter frequency effects such as propeller rotation rate to fluid momentum effects. By effects here is meant either forces or energies. As such, the Strouhal number is a dimensionless meter factor.

Conventionally, it is fluid mechanical practice to formulate the Reynolds number as the ratio of fluid inertial to viscous effects,

$$Re=\frac{D_{M0}U_{M0}}{\upsilon_{0}}.$$ (44)

This is generally used to describe the domain of the meter calibration for which the corresponding Strouhal numbers specify the range of meter response. A Strouhal-Reynolds characterization of a pulse-producing flowmeter conforms to conventional fluid mechanical procedures and it is analogous to orifice metering practice where discharge coefficient (a ratio of fluid inertia to differential pressure effects) is described functionally or graphically versus Reynolds number. However, as noted in Eq. (44) the Reynolds number requires that the fluid velocity be known. Since this is the purpose for using the meter, an iteration technique is required to calculate the flowrate. To avoid such an iteration, turbine meter manufacturers have designed their products to have high levels of linearity over wide flowrate ranges. As a result of this, it has been conventional turbine meter practice to characterize performance via *K*-factor vs. frequency to kinematic viscosity ratio—the so-called Universal Viscosity Curve (UVC). In accord with the principles of dimensional similitude, the meter frequency to fluid viscous effects can be formulated using, as characteristic length scale, the meter diameter, *D*_M0_ as in Eq. (44). This formulation can also be achieved via the product of Strouhal and Reynolds numbers; this product has been recently referred to, see [9], as the Roshko number,

$$R_{0}=\frac{f_{M0}D_{{M0}^{2}}}{\upsilon_{0}}.$$ (45)

This Roshko number is the dimensionless version of the frequency-to-kinematic viscosity ratio used for the UVC. It is expected that the dimensionless version of the UVC—the Strouhal-Roshko characterization—should produce superior prediction of meter performance compared to the UVC by virtue of its more complete, i.e., dimensionless description of the inertial, viscous, and oscillatory effects that occur in the meter operation.

**Fig. 7:**
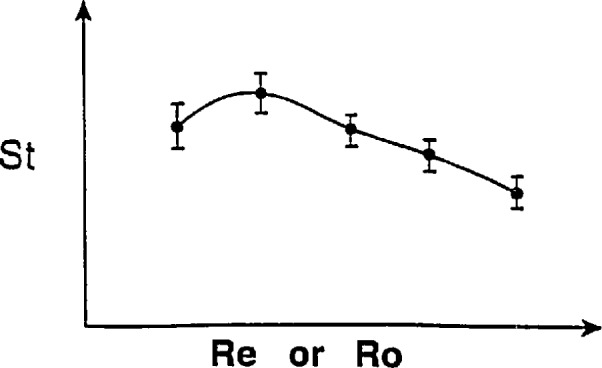
Normalized calibration results for a turbine-type flowmeter. Points denote averaged results; bars denote standard deviations obtained at each flowrate.

The question of which set of parameters best describes a turbine meter’s performance should be determined using appropriate data sets. The specific design considerations of blade size and shape, internal meter geometry, bearing features, etc., can be expected to play significant roles in the selection of non-dimensional parameters. Where it may happen that the Reynolds number produces a better description of turbine meter performance, i.e., the data collapses better onto a single curve than achieved using the Roshko number, an iterative sequence of computations may be necessary to produce an accurate determination of flowrate. If the meter is very linear, such an iteration may not be required. Typical flowmeter characterization results are sketched in Fig. 7. Because it is not known whether Reynolds or Roshko number is the better parameter with which to characterize the meter performance, both shall be included in what follows.

In those instances where meter performance might deviate from the curve shown in Fig. 7, the interpretation would be that additional factors in the deviant conditions have become significant whereas these factors were insignificant in the calibration conditions. Examples might be fluid frictional effects in the turbine bearings produced by extreme viscosity variations from those prevailing in the calibration conditions, or liquid cavitation effects, etc.

The curve shown in Fig. 7 is interpreted as the functional relationship between the Strouhal and Reynolds or Roshko numbers which are assumed to be the salient parameters describing the performance of this flowmeter over these calibration conditions. Inherent in this interpretation is the assumption that in any subsequent use of this functional relationship, the geometries of this meter, for example, the bearings or the propeller diameter are not changed relative to the selected characteristic length of the meter, i.e., the internal diameter, *D.* If such changes do occur it can be expected that the curve shown in Fig. 7 can change. For example, if a smaller propeller were installed, this curve may retain its shape but lie below that shown in Fig. 7. A non-dimensional parameter which could take into account different propeller diameters is *ß=d/D*, where *d* is the propeller diameter. Accordingly, the curve shown in Fig. 7 would pertain to the specific *β* for which the calibration was done. If other, smaller propellers were also calibrated, these results could be plotted in Fig. 7 and parameterized with the smaller value of *β.* When the propeller and meter-body materials are the same and where pressure effects can be neglected, the *β* ratio will remain constant when the temperature changes. When the propeller and the meter-body materials are different, temperature changes can produce different *β* ratios. These different ratios can be computed using pertinent relationships. The computed results should then be used with calibration data taken for different *β* ratios to predict the meter performance at the different temperature conditions, see Appendix B.

Figure 7 is different from conventional turbine meter performance plots in which the meter’s *K*-factor is plotted versus the ratio of frequency-to-kinematic viscosity. These conventional quantities are different from the Strouhal and Reynolds or Roshko number parameters by constant factors and by factors of the meter diameter raised to different exponents. These powers of the diameter should change only slightly with small changes in temperature and internal pressure. However, when conditions vary widely, the dimensionless formulations should be used and are expected to produce improved meter performance. Plots in the format of Fig. 7 should then, with the exceptions of deviant phenomena becoming influential, apply to a wide range of specific, dimensional fluid property and flow conditions and produce accurate predictions of turbine meter performance.

Where conventional turbine meter practice is used and plots are produced for K-factor versus the ratio of frequency-to-kinematic viscosity, improved meter performance can be expected when these calibration results are corrected to specified reference conditions. Accordingly, the corrected results should incorporate temperature and internal pressure corrections for the ratios of meter diameters raised to the relevant exponent to predict meter performance for other fluid and flow conditions. For meters having good linearity characteristics, i.e., constancy of the meter factor over specified flowrate ranges, the more important of these two corrections is that for the *K*-factor, i.e.,

$$K_{M0}=K_{M}{\left(D_{M}/D_{M0}\right)}^{3}.$$ (46)

This stipulates that the meter frequency-to-fluid inertial effects ratio, i.e., Strouhal number be the same in the actual conditions as in the reference conditions. To first order in temperature and pressure, separately, the diametral ratio can be written,

$$\begin{array}{l} {\left(\frac{D_{M}}{D_{M0}}\right)}^{3}=[1+3\alpha_{M}(T_{M}-T_{M0})]\\ \times \left[1+\frac{3(P_{M}-P_{M0})D_{M0}}{2t_{M0}E_{M}}\right]=\frac{K_{M0}}{K_{M}}, \end{array}$$ (47)

where *D*_M_ and *D*_M0_ are, respectively, the meter diameters at the non-reference conditions, *T*_M_ and *P*_M_ and reference conditions, *T*_M0_ and *P*_M0_. The quantities α_M_ and *E*_M_ are, respectively, the linear expansion coefficient and the modulus of elasticity of the meter body material for these conditions, and *t*_M0_ is the thickness of the meter body at reference conditions. The more significant of the two correction factors is usually that for temperature deviations from reference conditions. When the pressure correction can be neglected,

$$K_{M0}=K_{M}[1+3\alpha_{M}(T_{M}-T_{M0})],$$ (48)

or, to first order:

$$K_{M}=K_{M0}[1-3\alpha_{M}(T_{M}-T_{M0})].$$ (49)

This relationship duplicates that given in [10].

### 3.2 Non-Reference Conditions

**Fig. 8:**
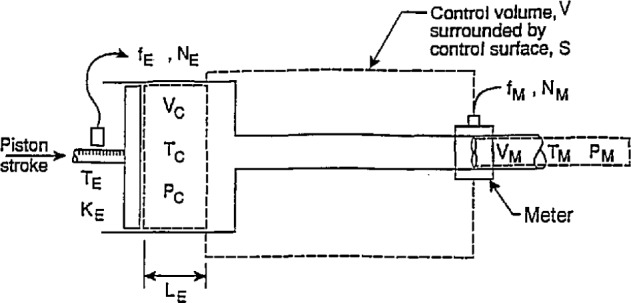
Arrangement for using characterized calibrator to calibrate a turbine-type flowmeter at non-reference conditions.

To calibrate a turbine-type flowmeter using the calibrator characterized as described above, the arrangement is sketched in Fig. 8. The temperature and pressure in the calibrator are the non-reference conditions denoted by *T*_C_ and *P*_C_. Applying again the conservation of mass principles, and assuming that there is no change of mass within the control volume with time, Eq. (11) and (12) indicate that

$$\rho_{C}{\overset{•}{V}}_{C}=\rho_{M}{\overset{•}{V}}_{M}\cdot$$ (50)

Since the fluid conditions in the cylinder and in the meter can be different, the ratio of the densities is

$$\frac{\rho_{C}}{\rho_{M}}=[1-3\alpha_{F}(T_{C}-T_{M})]\left[1+\frac{P_{C}-P_{M}}{E_{F}}\right],$$ (51)

and from Eq. (32) and in a manner analogous to Eq. (40)

$$\begin{array}{l} K_{C}=\frac{K_{E}}{{\bar{A}}_{C}}=\frac{f_{E}}{{\overset{•}{V}}_{C}}=\\ K_{C0}\frac{\left[1-\alpha_{E}(T_{E}-T_{0})\right]}{[1+2\alpha_{C}(T_{C}-T_{0}]\left[1+\frac{(P_{C}-P_{0})D_{C0}}{t_{C0}E_{C}}\right]}. \end{array}$$ (52)

Then, analogously to Eq. (42)

$$\begin{array}{l} K_{M}=\frac{f_{M}}{f_{E}}K_{C}\frac{\rho_{M}}{\rho_{C}}=\frac{N_{M}}{N_{E}}K_{C0}\\ \times \frac{\left[1-\alpha_{E}(T_{E}-T_{0})][1+3\alpha_{F}(T_{C}-T_{M})\right]}{[1+2\alpha_{C}(T_{C}-T_{0})]\left[1+\frac{(P_{C}-P_{0})D_{C0}}{t_{C0}E_{C}}\right]\left[1+\frac{\left(P_{C}-P_{M}\right)}{E_{F}}\right]}, \end{array}$$ (53)

where *K*_M_ has units of pulses per volume at the specific, non-reference meter conditions.

As stated above after Eq. (41), the pulse totals from the meter and the encoder have to correspond to the same displaced volume or time interval. If compensations are needed to achieve this correspondence, these should be done and results used in Eq. (53).

By normalizing this meter factor, *K*_M_ and the fluid flowrate using the non-reference meter diameter, *D*_M_, and fluid kinematic viscosity, υ, we obtain Strouhal and Reynolds or Roshko numbers

$$St=f_{M}D_{M}/U_{M}=C_{1}K_{M}D_{M}^{3},$$ (54)

where *C*_1_=π/4, and

$$Re=D_{M}U_{M}/\upsilon$$ (55)

or

$$Ro=\frac{f_{M}D_{M}^{2}}{\upsilon }.$$ (56)

Using these dimensionless parameters, the performance for the meter can be plotted; results should be as shown in Fig. 7. As noted above in Eqs. (43)–(45), the characteristics of meter factor and diameter at reference and non-reference conditions are interrelated and the dependence of the fluid’s kinematic viscosity can be written functionally as

$$\upsilon =\upsilon_{0}[T,T_{0},P,P_{0}].$$ (57)

With the performance curve given in Fig. 7 and the relationships given in Eqs. (43)–(45), or (54)–(57), one is now ready to use the flowmeter to make a flowrate measurement.

## 4. Using a Turbine-Type Flowmeter To Make a Measurement

### 4.1 Reference Conditions

**Fig. 9:**
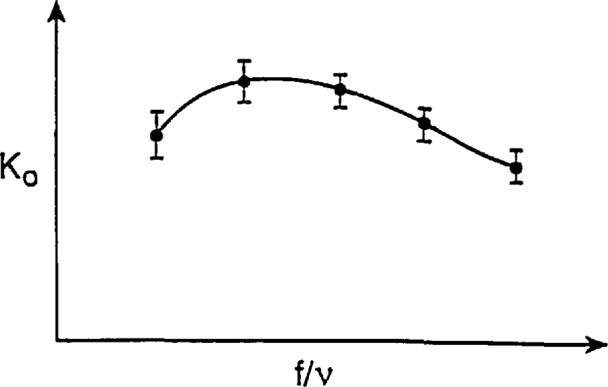
Conventional performance plot for a turbine-type flowmeter.

Given that the meter performance characteristics are as shown in Fig. 7, or less preferably but more conventionally as in Fig. 9, one can quantify the meter linearity over a specified flowrate range. The meter linearity is conventionally the average of the maximum and minimum values of the meter factor (Strouhal number) over this range; normalized by the average meter factor, see [3 and 10]. For the meter performance shown in Fig. 7, the mean value of the Strouhal Number, $\bar{St}$ gives the mean meter factor, via

$${\bar{K}}_{M0}=\frac{\bar{St}}{C_{1}D_{M0}^{3}},$$ (58)

where *C*_1_=π/4. The flowrate is determined via

$${\overset{•}{V}}_{M0}=\frac{f_{M0}}{{\bar{K}}_{M0}}=\frac{C_{1}D_{M0}^{3}f_{M0}}{\bar{St}}.$$ (59)

This result would pertain to any flowrate over the range specified for the meter’s linearity.

If it is desired to improve the accuracy of this flowrate determination, this could be done by using, for example, the curve shown in Fig. 7 or close approximations to it. With advances in today’s computer technology, this type of process can be readily installed in the secondary devices used with flowmeters. For the specific frequency from the meter, the Roshko number can be calculated directly and then used to determine the corresponding Strouhal number. For the case where the meter is characterized using Strouhal and Reynolds parameters, the process to determine an accurate flowrate should be iterative. This iteration process should begin using a mean value of meter factor, such as given in Eq. (58), this value of meter factor enables a computation of the flowrate via Eq. (59). Using this flowrate, the Reynolds number can be computed and then used to get the corresponding Strouhal number from the calibration curve and a refined value of flowrate. This process should be repeated until satisfactorily small changes are found in successive results. In this way, the accuracy level for the flowrate determination can be increased over the level associated with the meter’s linearity. This can be done to the precision level associated with a specific flowrate as quantified in the calibration process. The resulting enhanced meter performance could amount to significant improvements in measurement accuracy. In the following, a Strouhal-Reynolds characterization of meter performance will be used since it is more conventional in fluid mechanics and since it may require the iteration procedure, described above, to be used.

### 4.2 Non-Reference Conditions

To describe meter performance in non-reference conditions, the meter characteristics shown in Fig. 7 will be used. The reason for this is that the complete, non-dimensional assessment of meter frequency effects and fluid inertial and viscous effects are not complete in Fig. 9. For this reason, the non-reference meter diameter, *D*_M_, and frequency,*f*_M_, and the fluid’s kinematic viscosity, *v*, should be used, in compatible units, to produce the Reynolds number for the non-reference conditions. This Reynolds number produces, using Fig. 7, the corresponding Strouhal number which with the meter diameter, *D*_M_, gives the meter factor, *K*_M_-The flowrate measurement is then obtained using

$${\overset{\cdot }{V}}_{M}=\frac{f_{M}}{{\bar{K}}_{M}}=\frac{C_{1}f_{M}D_{M}^{3}}{\bar{St}}.$$ (60)

This flowrate is correctly converted to reference conditions by specifying that the Strouhal number is the same for the non-reference and reference conditions, specified by this Reynolds number, i.e.,

$$St=C_{1}\frac{f_{M}D_{M}^{3}}{{\overset{\cdot }{V}}_{M}}=C_{1}\frac{f_{M0}D_{M0}^{3}}{{\overset{\cdot }{V}}_{M0}}.$$ (61)

Therefore

$${\overset{\cdot }{V}}_{M0}={\overset{\cdot }{V}}_{M}\frac{f_{M0}}{f_{M}}{\left(\frac{D_{M0}}{D_{M}}\right)}^{3}.$$ (62)

The reference to non-reference frequency ratio is obtained by specifying that Reynolds number similarity exists for these two conditions, i.e.,

$$Re=\frac{f_{M}D_{M}^{2}}{(St)\upsilon_{M}}=\frac{f_{M0}D_{M0}^{2}}{(St)\upsilon_{M0}}.$$ (63)

It is noted that this is equivalent to stipulating that Roshko number similarity exists for these two conditions. From Eq. (63)

$$\frac{f_{M0}}{f_{M}}=\frac{\upsilon_{M0}}{\upsilon_{M}}{\left(\frac{D_{M}}{D_{M0}}\right)}^{2}$$ (64)

and

$${\overset{\cdot }{V}}_{M0}=\frac{C_{1}f_{M}D_{M0}^{3}}{St}\left(\frac{\mu_{M0}}{\mu_{M}}\right)\frac{[1+2\alpha_{M}(T_{M}-T_{M0})]\left[1+\frac{(P_{M}-P_{M0})D_{M0}}{t_{M0}E_{M}}\right]}{[1+3\alpha_{F}(T_{M}-T_{M0})]\left[1-\frac{\left(P_{M}-P_{M0}\right)}{E_{F}}\right]}.$$ (65)

It is apparent that, to obtain high accuracy flowrate measurements using the procedures described above, appropriately high accuracy measurements are required for the component measurement systems and for the pertinent material properties as shown in Eq. (65). In turn, it appears feasible that once systematic uncertainties are satisfactorily removed from calibration facilities, the measurement processes in calibration laboratories, and the measurement systems making on-line measurements will be commensurately improved and the uncertainty levels for these measurements can be predicted using such Eqs. as (2), (10), (25), and (65).

## 5. Discussion

The above-derived results can, for the sake of brevity, be assessed by considering the effects of temperature and pressure on the respective factors–the calibrator and flowmeter constants and the flowrate measurement at reference conditions. To do this Table 1 shows, for specific material conditions and geometrical sizes, the variations associated with temperature and pressure effects separately and then summed together.

The results shown in the first row of Table 1 present the variations in calibrator constant *K*_C0_ determined using geometrical measurement methods for variations of ± 1 °C in temperature and ±l×l0^5^Pa (1 atm) in pressure. The material properties and geometrical assumptions are given under the headings of the respective columns. The worst-case combination of temperature and pressure variations taken separately are given in columns 7 and 12, respectively. These results are obtained by adding the absolute values of the component contributions. The total worst-case combination for temperature and pressure variations taken together is given in the column at the right side of the table. Accordingly, the total temperature effect on *K*_C0_ in the measurement method is five times larger than the pressure effect and the total of these gives an imprecision of ± 0.006%.

The results for *K*_C0_ determined using the draw technique are shown in the second row. These give a total temperature variation of ±0.095% owing mainly to fluid expansion effects. The pressure variation is noted to be a factor of about 16 less than this level and the total imprecision totals ±0.101% which is about a factor of 20 larger than the level achieved using the geometrical measurement technique.

The results for the meter factor, *K*_M0_, are given in the third row of the table. Here, at reference conditions, no additional uncertainties are shown over those for *K*_C0_ in accord with Eq. (42).

The fourth row indicates that the total temperature variation for *St* is increased over that for *K*_M0_ by the amount allocated for meter expansion. This produces the ±0.101% uncertainty total for temperature which when added to the increased pressure total gives ±0.108%. This shows that while the Strouhal number is the preferred non-dimensional parameter to characterize the frequency effects of a meter, it has more factors than the conventional meter factor, *K*_M0_ and, therefore, it can have increased uncertainty. However, because the Strouhal number is a dimensionless ratio of frequency to inertial effects in the meter it should be successful in producing more satisfactory metering results for widely ranging conditions than can be done using a dimensional quantity such as *K*_M0._

The fifth and sixth rows express the uncertainties for *K*_M_ and the corresponding Strouhal number in a manner analogous to that used to obtain values in rows three and four. However, as shown in Eq. (53) the uncertainties attributed to *K*_C0_ are increased appropriately due to encoder, calibrator, and fluid effects; and, in the case for *St*, meter effects. It is noted that the totaled uncertainties have essentially doubled in comparison with those for *K*_M0_.

While it is recognized that the conditions of ± 1 °C and ± 1 × 10^5^ Pa (1 atm) pressure variation can be termed large, it should be recognized that no uncertainty has been allocated in Table 1 for the uncertainty with which the material properties and geometrical dimensions are known or have been determined. As well, it should be recognized that these assumed conditions of ± 1 °C and ± 1×10^5^ Pa (1 atm) can in reality understate the actual variations that may exist in real situations where flowrate measurements can be attempted in harsh, hostile environments.

It should be emphasized that the above discussion deals only with the uncertainties associated with temperature and pressure imprecision and does not take into account contributions from flowmeter imprecision such as the variability of detecting meter pulses. Additionally, systematic errors which can greatly exceed the levels of imprecision are not included in any of the above.

A summary of the equations derived for characterizing piston-type, encoded-stroke calibrators, their calibration procedures, and the subsequent use of calibrated turbine meters is given in Appendix C. These equations are found to be quite simple when reference conditions prevail. However, reference conditions are practically fictitious and therefore the performance of all of these devices should be considered as occurring in non-reference conditions. Given the capabilities available in today’s computers, it is felt that the complete equations for these types of devices should be installed in the controlling and processing software so that when temperature and or pressure conditions become significant in these processes the results are accurate. Using these equations when temperature and or pressure effects are not significant produces negligible differences from the results at reference conditions.

## 6. Conclusions

High accuracy fluid measurements can be attained when the important factors affecting the performances of both flowmeter calibrators and fluid meters are properly taken into account in the measurement processes of these devices. To do this–to first order temperature and pressure effects on both the calibrator and the meter as well as on the fluid–the equations derived above should be used as the basic system models for calibrator and meter. Specific features of calibrators need to be analyzed and, where pertinent, appropriate modifications to the above-derived equations should be made. Calibrators and metering units need to be properly instrumented and operated according to the required assumptions and techniques for handling the data. Where further increases in flow measurement accuracy are needed, either more accurate descriptions of currently considered factors should be made or additional factors not currently considered should be assessed and included as pertinent, or both.

## Figures and Tables

**Table 1:** Temperature and pressure effects

Factor affected	Total diff (°C)	Temperature effects				Total temperature combined worst case	Press diff (atm)	Pressure effects			Total pressure combined worst case	Total worst case
		Encoder factor (α_E_ = 10^−5^°C^−1^)	Calibrator cross-section (*α*_c_=2×10^−5^ °C^−1^)	Fluid expansion (*α*_F_=3×10^−4^°C^−1^)	Meter expansion (α_M_=2×10^−5^ °C^−1^)			Calibrator cross-section (*D*/*t*=20; *E*_c_=2×10^11^Pa)	Fluid expansion (*E*_F_=2×l0^9^Pa)	Meter expansion (*D*/*t*=15; *E*_M_=2×l0^11^Pa)		
*K*_C0:GEOM_^a^	±1	±0.001%	±0.004%			±0.005%	±1	±0.001%			±0.001%	±0.006%
*K*_C0:DRAW_^b^	±1	±0.001%	±0.004%	±0.090%		±0.095%	±1	±0.001%	±0.005%		±0.006%	±0.101%
*K*_M0_^c^	±1	±0.001%	±0.004%	±0.090%		±0.095%	±1	±0.001%	±0.005%		±0.006%	±0.101%
*St*^d^	±1	±0.001%	±0.004%	±0.090%	±0.006%	±0.101%	±1	±0.001%	±0.005%	±0.001%	±0.007%	±0.108%
*K*_M_^e^	±1	±0.002%	±0.008%	±0.180%		±0.190%	±1	±0.002%	±0.010%		±0.012%	±0.202%
*St*^f^	±1	±0.002%	±0.008%	±0.180%	±0.006%	±0.196%	±1	±0.002%	±0.010%	±0.001%	±0.013%	±0.209%
${\overset{*}{V}}_{\mathrm{MU}}$^g^	±1	±0.001%	±0.004%	±0.090%		±0.090%	±1	±0.001%	±0.005%		±0.006%	±0.101%
${\overset{*}{V}}_{M}$^h^	±1	±0.002%	±0.008%	±0.180%		±0.190%	±1	±0.002%	±0.010%		±0.012%	±0.202%
${\overset{*}{V}}_{\mathrm{MU}}$^i^	±1	±0.002%	±0.008%	±0.270%	±0.012%	±0.292%	±1	±0.002%	±0.015%	±0.002%	±0.019%	±0.311%

a Determined via Eq. (10) at non-reference conditions.

b Determined via Eq. (29) at non-reference conditions with no additional uncertainty for the encoder or the collection volume.

c Determined using *K*_CU:DRAW_ via Eq. (42) with no additional uncertainty resulting from encoder or meter outputs.

d Determined using *K*_CU:DRAW_ via Eq. (43) in reference conditions.

e Determined using *K*_CU:DRAW_ via Eq. (53).

f Determined using *K*_CU:DRAW_ via Eq. (54).

g Determined using *K*_M0_ via Eq. (59) with no additional uncertainty resulting from the meter output.

h Determined using *K*_M_ via Eq. (60) with no additional uncertainty resulting from meter output.

i Determined using Eq. (65) with not additional uncertainty for meter output, reference meter dimension, and absolute viscosity.

**Fig. A.2.1:**
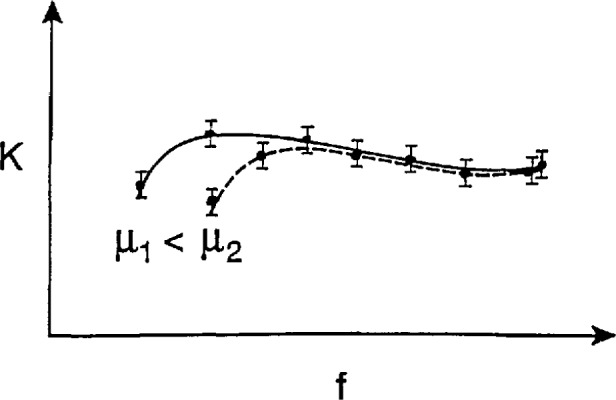
Conventional format for processing turbine-type flowmeter data.

**Fig. A.2.2:**
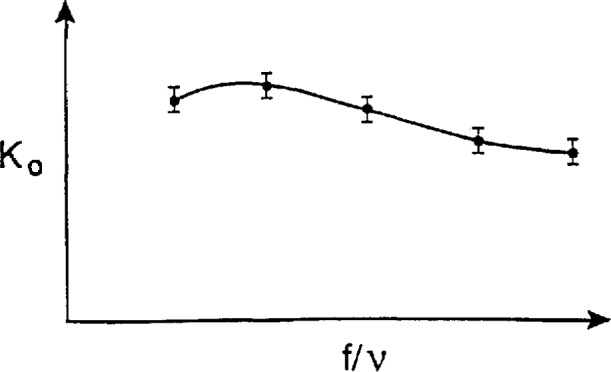
Universal Viscosity Curve (UVC) for processing turbine-type flowmeter data.

**Fig. A.2.3:**
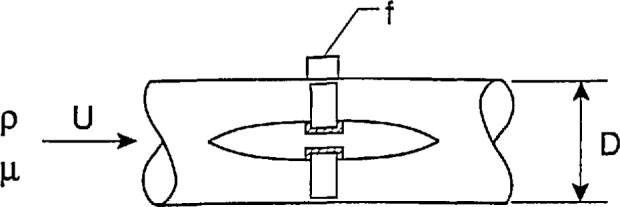
Turbine meter configuration.

**Fig. A.2.4:**
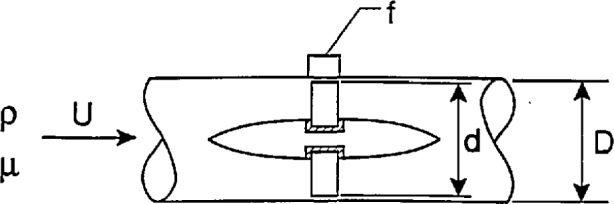
Turbine meter configuration with the turbine wheel diameter, *d*, considered as a parameter.

**Fig. A.2.5:**
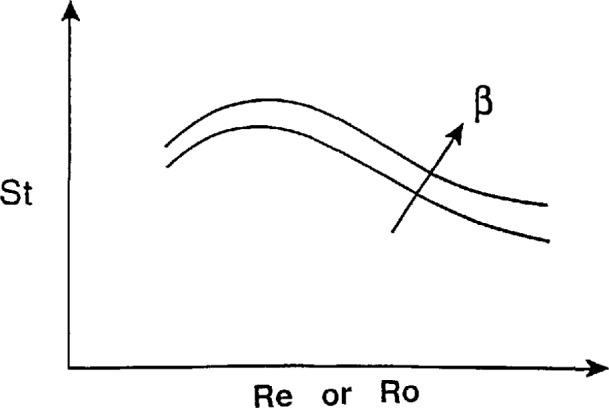
Non-dimensional turbine meter performance.

**Fig. A.2.6:**
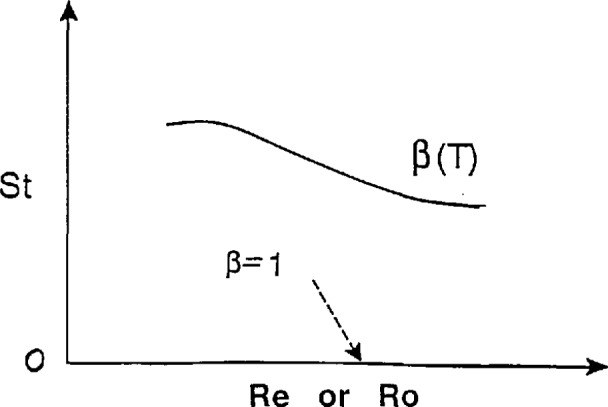
Sketch of expected turbine meter performance where *β*→1.

## Glossary

**Table:**

*Symbol*	*Description*	*Dimensions*
*A*_C0_, *A*_C_	Calibrator cross-sectional areas at reference and non-reference conditions, respectively. Over-bars, where used, denote averages over piston stroke length.	L^2^
*A*_t0_, *A*_t_	Cross-sectional areas of tubes connected to calibrator piston at reference and non-reference conditions, respectively.	L^2^
*C*_0_, *C*	Circumference of cylindrical body in unstressed and stressed conditions, respectively.	L
*d*_0_, *d*	Diameter of rod connected to calibrator piston at reference and non-reference conditions, respectively.	L
*D*_0_, *D*	Inner diameter of calibrator cylinder at reference and non-reference conditions, respectively.	L
*D*_M0_, *D*_M_	Inside diameter of flowmeter at reference and non-reference conditions, respectively.	L
*d*_M0_, *d*_M_	Outside diameter of turbine propeller at reference and non-reference conditions, respectively.	L
*D*_C0_, *D*_C_	Inside diameter of the cylinder of the calibrator at reference and non-reference conditions, respectively.	L
*D*_T0_, *D*_T_	Outside diameter of the tube connected to the calibrator piston at reference and non-reference conditions, respectively.	L
*E*_C_	Modulus of elasticity of the material of the calibrator cylinder at pertinent conditions.	F/L^2^
*E*_F_	Modulus of elasticity of the fluid at pertinent conditions.	F/L^2^
*E*_M_	Modulus of elasticity of the material of the body of the flowmeter at pertinent conditions.	F/L^2^
*E*_T_	Modulus of elasticity of the material of the tube connected to the calibrator piston at pertinent conditions.	F/L^2^
*f*_E0_, *f*_E_	Frequency of pulses from encoder at reference and non-reference conditions, respectively.	pulses/t
*f*_M0_, *f*_M_	Frequencies of pulses from the flowmeter at reference and non-reference conditions respectively.	pulses/t
*K*_E0_, *K*_E_	Encoder constant at reference and non-reference conditions, respectively.	pulses/L
*K*_C0_, *K*_C_	Calibrator constant at reference and non-reference conditions, respectively.	pulses/L^3^
*K*_Ć_	Calibrator constant in pulses per fluid volume collected in collection vessel.	pulses/L^3^
*K*_M0_, *K*_M_	Meter factor at reference and non-reference conditions, respectively. Where over-bars are used is meant the average value over a Reynolds number range.	pulses/L^3^
*L*_E0_, *L*_E_	Piston stroke length at reference and non-reference conditions, respectively.	L
*N*_E0_, *N*_E_	Totalized encoder pulses at reference and non-reference conditions, respectively.	pulses
*N*_M0_, *N*_M_	Totalized meter pulses at reference and non-reference conditions, respectively.	pulses
***n***	Unit vector.	dimensionless
*P*_C0_, *P*_C_	Pressure of the fluid in the cylinder of the calibrator at reference and non-reference conditions, respectively.	F/L^2^
*P*_COLL’T_	Pressure of fluid in collection vessel.	F/L^2^
*P*_C0_, *P*_C_	Pressure of the fluid in the flowmeter at reference and non-reference conditions, respectively.	F/L^2^
*Re*	Reynolds number.	dimensionless
*Ro*	Roshko number.	dimensionless
*St*	Strouhal number. Where over-bars are used is meant the average value over a Reynolds number range.	dimensionless
*S*	Surface area of control volume.	L^2^
*T*_r_	Temperature of meter rotor or of rod connected to calibrator piston.	°C (°F)
*T*_C0_, *T*_C_	Temperature of the cylinder of the calibrator at reference and non-reference conditions, respectively.	°C (°F)
*T*_E0_, *T*_E_	Temperature of the encoder at reference and non-reference conditions, respectively.	°C (°F)
*t*_C0_	Thickness of the cylinder of the calibrator at reference conditions.	L
*t*_T0_	Thickness of the tube connected to the to the calibrator piston at reference conditions.	L
*t*	Time.	t
*T*_COLL’T_	Temperature of fluid in collection vessel.	°C (°F)
*T*_M0_, *T*_M_	Temperature of the flowmeter at reference and non-reference conditions, respectively.	°C(°F)
*t*_M0_	Thickness of the body of the flowmeter at reference conditions.	L
*U*_M0_	Average velocity of fluid through the flowmeter at reference conditions.	L/t
*V*_C0_, *V*_C_	Calibrator piston displacement at reference and non-reference conditions, respectively.	L^3^
***v***	Fluid velocity vector.	L/t
*V*	Control volume.	L^3^
${\dot{V}}_{C0}$, *V*_C0_	Fluid volumetric flowrate and volume, respectively, in the calibrator cylinder at reference condition.	L^3^/t,L^3^
${\dot{V}}_{{\mathrm{COLL}}^{’}T0}$, *V*_COLL’TO_	Fluid volumetric flowrate and volume, respectively, into collection vessel at reference conditions.	L^3^/t,L^3^
${\dot{V}}_{C}$, *V*_C_	Fluid volumetric flowrate and volume, respectively, in the calibrator cylinder at non-reference conditions.	L^3^/t,L^3^
${\dot{V}}_{M0}$, *V*_M0_	Fluid volumetric flowrate and volume, respectively, in flowmeter at reference conditions.	L^3^/t,L^3^
${\dot{V}}_{M}$, *V*_M_	Fluid volumetric flowrate and volume, respectively, through the flowmeter at non-reference conditions.	L^3^/t,L^3^
*α*_E_	Linear expansion coef-of the encoder at pertinent conditions.	°C^−1^(°F^−1^)
*α*_C_	Linear expansion coefficient for the material of the calibrator cylinder at pertinent conditions.	°C^−1^(°F^−1^)
*α*_F_	Linear expansion coefficient of fluid.	°C^−1^ (°F^−1^)
*α*_M_	Linear expansion coefficient for the material of the body of the flowmeter.	°C^−1^ (°F^−1^)
*α*_r_	Linear expansion coefficient for the material of rotor or turbine wheel.	°C^−1^ (°F^−1^)
*β*	Ratio of turbine propeller diameter to inside diameter of meter.	dimensionless
ϵ_θ_	Hoop strain in cylindrical body.	dimensionless
*ρ*	Fluid density.	M/L^3^
*ρ*_C0_, *ρ*_C_	Fluid density in calibrator at reference and non-reference conditions, espectively.	M/L^3^
*ρ*_COLL’T0,_ *ρ*_COLL’T_	Density of fluid collected in collection vessel at reference and non-reference conditions, espectively.	M/L^3^
*ρ*_M0_, *ρ*_M_	Density of the fluid in the flowmeter at reference and non-reference conditions, respectively.	M/L^3^
σ_θ_	Hoop stress in cylindrical body.	F/L^2^
ν_0_, *v*	Fluid kinematic viscosity at reference and non-reference conditions, espectively.	L^2^/t
*μ*_0_, *μ*	Fluid absolute viscosity at reference and non-reference conditions, espectively.	Ft/L^2^

## 7. Appendix A. Cross-Sectional Area Changes in the Cylinder of the Calibrator

The fluid pressure and temperature inside the cylinder of the calibrator can enlarge or reduce the cross-sectional areas of this cylinder, see Fig. 1. As well, geometrical changes of the rod or tube connected to the piston can also contribute to changes in this cross-sectional area. Of course, knowledge of specific geometries and material properties are crucial to characterizing these changes. In the following it is assumed that a generic cylinder and rod geometry exist as sketched in Fig. 1.

First, thermal expansion will increase the diameters of the cylinder and the connecting tube or rod via the first order approximations

$$D=D_{0}[1+\alpha_{C}(T_{C}-T_{0}]$$ (A.1.1)

and

$$d=d_{0}[1+\alpha_{r}(T_{r}-T_{0}],$$ (A.1.2)

where, in compatible units, *D* and *d* and *D*_0_ and *d*_0_ are the cylinder and tube diameters at non-reference and reference conditions, respectively. The linear expansion coefficients for cylinder and tube or rod are *α*_C_ and *α*_r_, respectively, and *T*_C_ and *T*_r_ are the corresponding temperatures. These diametral enlargements will produce an increased cross-sectional area via the first order approximation

$${\bar{A}}_{C}={\bar{A}}_{C0}[1+2\alpha_{C}(T_{C}-T_{0}],$$ (A.1.3)

where *Ā*_C_ and *Ā*_C0_ are, respectively, the annular areas between cylinder and rod as averaged over the appropriate piston displacement. The linear expansion coefficient of the rod or tube is assumed to be the same as that for the cylinder.

Second, pressure effects can change the cross-sectional area through the enlargement of the calibrator cylinder and the contraction of the rod or tube connected to the piston. The azimuthal or “hoop” stress, *σ_β_*, produced in the calibrator cylinder can be shown to be

$$\sigma_{\theta }=(P_{C}-P_{0})D_{C}/2t_{C},$$ (A.1.4)

where *P*_C_–*P*_0_ is the pressure difference between the pressure in the cylinder and the reference pressure outside the cylinder. The quantities *D*_C_ and *t*_C_ are, respectively, the inside diameter and the thickness of the cylinder. The azimuthal strain produced by this stress is assumed to be

$$\in_{\theta }=\sigma_{\theta }/E_{C},$$ (A.1.5)

where transverse effects are neglected and where *E*_C_ is the modulus of elasticity of the material of the calibrator. This azimuthal strain increases the circumference of the calibrator cylinder via

$$C=C_{0}(1+\in_{\theta }),$$ (A.1.6)

where *C* and *C*_0_ are, respectively, the inside circumferences of the cylinder in stressed and unstressed conditions. This produces an increase, to first order, in the cylinder’s averaged cross-sectional area of

$${\bar{A}}_{C}=\frac{{\bar{C}}^{2}}{4\pi }=\frac{{{\bar{C}}_{0}}^{2}}{4\pi }\left[1+\frac{(P_{C}-P_{0})D_{C0}}{t_{C0}E_{C}}\right]$$ (A.1.7)

or since ${\bar{A}}_{C0}={{\bar{C}}_{0}}^{2}/4\pi ,$

$${\bar{A}}_{C}={\bar{A}}_{C0}\left[1+\frac{(P_{C}-P_{0})D_{C0}}{t_{C0}E_{C}}\right],$$ (A.1.8)

where *D*_C0_ and *t*_C0_ are the diameter and thickness, respectively, of the calibrator cylinder at reference conditions. Typical values for the ratio *D*_C0_/*t*_C0_could be 10 to 20.

In similar fashion, the cylinder pressure in excess of the reference pressure, *P*_0_ at which condition the reference dimensions are determined can reduce the outside diameter of the tube connected to the piston. Since the deformation of a tube should, for the same material and diameter, exceed that for a solid rod, it shall be considered here. Therefore, the cross-sectional area of the tube connected to the piston would be

$${\bar{A}}_{t}={\bar{A}}_{t0}\left[1-\frac{(P_{C}-P_{0})D_{t0}}{t_{t0}E_{t}}\right],$$ (A.1.9)

where *D*_t0_ and *t*_t0_ are, respectively, the outside diameter and the thickness of the wall of the tube connected to the piston at reference conditions. The quantity *E*_T_ is the modulus of elasticity of the tube material, at reference conditions.

It is generally and safely assumed that the tube contraction effect expressed in Eq. (A.1.9) is negligible in comparison to the effects of temperature on the inner diameter of the calibrator. Therefore, it is assumed that the annular cross-sectional area between cylinder and tube is that expressed in Eq. (A.1.3) and the effect of pressure on this annular area is expressed in Eq. (A.1.8). However, where specifics indicate that other compensations need to be made, other approximations can and should be done. The combined effect of pressure and temperature on the averaged cross-sectional area of the cylinder of the calibrator is,

$${\bar{A}}_{C}={\bar{A}}_{C0}[1+2\alpha_{C}(T_{C}-T_{C0}]\left[1+\frac{(P_{C}-P_{0})D_{C0}}{E_{C}t_{C0}}\right].$$ (A.1.10)

## 8. Appendix B. Turbine Meter Data Processing

Conventional practice in processing and using turbine-type flowmeter data began with plotting the meter *K* factor in pulses per volume units as a function of meter frequency, *f*; see Fig. A.2.1 and [5–8]. In this figure, the bracketed dots are intended to represent the mean values and the scatter obtained for the *K* factors at respective frequencies. For fluid and flow conditions which precisely duplicate those for which the meter calibration was performed, this practice produced acceptable results. However, when fluid or flow conditions deviated from those of the calibration, it was found that results could be unacceptable. An example is indicated in Fig. A.2.1. The lines for which the liquid viscosities are *μ*_1_ and *μ*_2_, respectively, indicate that different meter performance occurs at low flowrates when the fluid dynamic viscosity, *μ*_2_ is greater than the value *μ*_1_ used for the calibration.

Flowmeter performance which deviates from that of the calibration conditions at low flowrates is explained by citing the deviant phenomena which have, because of the different fluid or flow conditions, become significant in comparison to the phenomena which prevailed during calibration. Shafer and Lee have resolved several situations involving viscous effects in specific flowmeter geometries [6,8]. From such efforts have come Universal Viscosity Curves (UVC) i.e., plots of meter factor versus the ratio of meter frequency to fluid kinematic viscosity. A typical UVC is shown in Fig. A.2.2. The ordinate, *K*_0_, is the meter factor referenced to a selected reference temperature via, see [10],

$$K_{0}=K[1+3\alpha_{M}(T-T_{0}].$$ (A.2.1)

Deviations of flowmeter performance characteristics from UVC’s can be explained by citing further deviants, such as lubricity, cavitation, extreme temperature or pressure effects on fluid properties, etc., see [5–8].

When the turbine meter is to be used in temperature conditions different from those of the reference or calibration conditions, temperature effects may be compensated for by producing corrections for the meter factor *K*, as found in some standards, see [10]. This ISA standard produces an underived correction that is based upon the thermal expansion for the material of the meter body. This correction is effective in predicting trends for the case where the meter body and the turbine wheel material are the same. Where these materials are different, this correction can give erroneous results, for example, in the extreme situation where the turbine wheel has expanded to touch the inner wall of the meter body, thus stopping the wheel’s rotation. In such a situation the meter factor should be zero yet the ISA correction would not give this result. Therefore, further evolution is needed for conventional turbine-type flowmeter practice—beyond the UVC methods.

Turbine meter performance is most properly characterized by specifying and quantifying, over the pertinent ranges of fluid and flow conditions, the significant dimensionless parameters which influence meter performance. To do this, the dimensional quantities that are involved in turbine-type flowmetering are cited as shown in Fig. A.2.3. These five dimensional quantities—fluid density *ρ*, and viscosity *μ*, the flow velocity *U*, the meter frequency *f*, and the meter diameter, *D* are described in terms of three independent units — length, time, and mass or force. Consequently, there are two dimensionless parameters to characterize the performance of this meter. These dimensionless parameters can take many forms. Conventional fluid mechanical practice would produce parameters expressing ratios of meter frequency effects to fluid inertial effects, i.e., the Strouhal number and the fluid inertial to viscous effects, i.e., the Reynolds number. These are written

St=fD/U (A.2.2)

and

$$Re=\frac{DU_{\rho }}{\mu }=\frac{DU}{\upsilon }.$$ (A.2.3)

However, turbine meters are designed to have frequencies proportional to volumetric flowrates. The constant of proportionality is the reciprocal of the meter factor. Since the constancy of this meter factor, i.e., the linearity of the meter over the flowrate (frequency) range is generally taken to be a measure of the quality of the meter, it has become conventional turbine meter practice to characterize meters via their *K* factor versus frequency dependence. In non-dimensional format, this can be done by specifying meter performance using a Strouhal number that is dependent upon the product of the Strouhal and Reynolds numbers, i.e., *fD*^2^/*v.* This parameterization is essentially that known as the UVC except for the factors involving the meter diameter, *D*, raised to an exponent. This product has been referred to as the Roshko number, *Ro*,

$$Ro=\frac{fD^{2}}{\upsilon }$$ (A.2.4)

see [9]. Alternatively, the pair of parameters, *St* and *Re* could also be used to characterize the meter’s performance. In either case, only two parameters are needed to characterize the system sketched in Fig. A.2.3.

In all of the above, the effect of different meter geometries or a different turbine wheel is not considered. This is proper where the meter geometry is not changed and where the same turbine wheel and bearings, etc. are used and where the turbine wheel and the meter body are made of the same material. Under these circumstances, the above parameterization of the five dimensional quantities *ρ,μ,U,D*, and *f* is completed with the formulation of the Strouhal and Reynolds or Roshko numbers. Using this formulation the performance of the calibrated meter should enable the meter to be satisfactorily used over a range of conditions similar to those encountered in the calibration.

If the situation is changed as shown in Fig. A.2.4 so that the turbine wheel diameter, *d*, is included to produce six dimensional quantities, then three dimensionless parameters should be formed. The turbine wheels that are considered here are geometrically scaled versions of each other, i.e., they have the same number of blades, the same blade shape and only differ in the diameter, *d.* The nondimensionalization can be achieved by choosing the third dimensionless parameter to be *β=d/D*, the ratio of turbine wheel to inside pipe diameter. The three parameter performance is sketched in Fig. A.2.5. It is noted that when the turbine wheel and meter body are the same material and when only thermal expansion effects are considered, *β* remains constant with changes in temperature, i.e.,

$$\beta =\frac{d}{D}=\frac{d_{0}[1+\alpha_{r}(T_{r}-T_{0}]}{D_{0}[1+\alpha_{M}(T_{M}-T_{0}]},$$ (A.2.5)

when *T*_r_ = *T*_M_ and *α*_r_ *= α*_M_ this diameter ratio is *d*_0_/*D*_0_.

When turbine wheel and meter body are different materials and when *α*_r_*>α*_M_, the beta ratio is not constant and a condition for the linear expansion coefficients and the fluid flowrate can be derived for which *T*_M_ *= T*_r_, and *β* = 1. This would indicate that the turbine blade tips touch the inside of the meter body thus stopping the wheel. This condition is, of course, contrived in order to illustrate the point that *β* variation can produce meter performance variation and in extreme limits can radically alter the graphs of meter performance.

If pressure effects are not negligible in altering the diameter of the meter body but are negligible in affecting the diameter of the turbine wheel, *β* is altered, to first order in temperature and pressure, separately, via

$$\beta =\frac{d_{0}[1+\alpha_{r}(T_{r}-T_{0})]}{D_{0}[1+\alpha_{M}(T_{M}-T_{0})\left[1+\frac{(P_{M}-P_{0})D_{M0}}{2t_{M0}E_{M}}\right]}.$$ (A.2.6)

By so incorporating thermal and pressure effects using *St*, *Re* or *Ro*, and *β* parameters into performance curves as sketched in Fig. A.2.6, widely varying metering conditions should be successfully handled.

It is concluded that turbine-type flowmeter performance should be handled non-dimensionally. Pertinent corrections are then most clearly seen and most easily performed using the appropriate, first-order, relationships for the thermal and pressure effects. In this way high levels of flowmeter performance can be expected and achieved.

## 9. Appendix C. Equations Summary

The equations in Appendix C are numbered as in the main text.

1. *Calibrator Characterization:*
  - Geometrical Measurement Method—Reference Conditions (see p. 513)
    $$K_{C0}=\frac{N_{E0}}{V_{C0}}=\frac{L_{E0}K_{E0}}{{\bar{A}}_{C0}L_{E0}}=\frac{K_{E0}}{{\bar{A}}_{C0}}.$$ (2)
  - Geometrical Measurement Method–Non-Reference Conditions (see p. 515)
    $$K_{C0}=K_{C}\frac{\left[1+\alpha_{E}(T_{E}-T_{0})\right]}{[1-2\alpha_{C}(T_{C}-T_{0}]\left[1-\frac{(P_{C}-P_{0})D_{C0}}{t_{C0}E_{C}}\right]},$$ (10)
    where
    $$K_{C}=\frac{N_{E}}{V_{C}},$$ (7)
  - Draw Technique–Reference Conditions (see p. 516)
    $$K_{C0}=N_{E0}/V_{{\mathrm{COLL}}^{’}T0}.$$ (15)
  - Draw Technique–Non-Reference Conditions (see p. 517)
    $$\begin{array}{l} K_{C0}=K_{C}^{\prime }\\ \frac{[1+2\alpha_{C}(T_{C}-T_{0})]\left[1+\frac{(P_{C}-P_{0})D_{C0}}{t_{C0}E_{C}}\right]}{\left[1-3\alpha_{F}(T_{{\mathrm{COLL}}^{’}T}-T_{C})]\left[1+\frac{P_{{\mathrm{COLL}}^{’}T}-P_{C}}{E_{F}}\right][1-\alpha_{E}(T_{E}-T_{0})\right]} \end{array}$$ (29)
    where
    $$K_{C}^{\prime }=N_{E}/V_{{\mathrm{COLL}}^{’}T}.$$ (28)
2. *Meter Calibration:*
  - *Reference Conditions:* (see p. 519)
    $$K_{M0}=\frac{f_{M0}}{f_{E0}}\;\frac{K_{E0}}{{\bar{A}}_{C0}}=\frac{N_{M0}}{N_{E0}}\;\frac{K_{E0}}{{\bar{A}}_{C0}}=\frac{N_{M0}}{N_{E0}}•K_{C0}=\frac{f_{M0}}{f_{E0}}•K_{C0}.$$ (42)
    *Data Processing*: (see p. 519)
    $$St=\frac{f_{M0}D_{M0}}{U_{M0}}=C_{1}K_{M0}D_{M0}^{3}\propto K_{M0}D_{M0}^{3},$$ (43)
    $$Re=\frac{D_{M0}U_{M0}}{\upsilon_{0}},$$ (44)
    $$R_{0}=\frac{f_{M0}D_{M0}^{2}}{\upsilon_{0}}.$$ (45)
  - *Non-Reference Conditions:* (see p. 521)
    $$\begin{array}{l} K_{M}=\frac{f_{M}}{f_{E}}K_{C}\frac{\rho_{M}}{\rho_{C}}=\frac{N_{M}}{N_{E}}K_{C0}\\ \times \frac{\left[1-\alpha_{E}(T_{E}-T_{0})][1+3\alpha_{F}(T_{C}-T_{M})\right]}{[1+2\alpha_{C}(T_{C}-T_{0})]\left[1+\frac{(P_{C}-P_{0})D_{C0}}{t_{C0}E_{C}}\right]\left[1+\frac{\left(P_{C}-P_{M}\right)}{E_{F}}\right].} \end{array}$$ (53)
    *Data Processing:* (see p. 522)
    $$St=f_{M}D_{M}/U_{M}=C_{1}K_{M}D_{M}^{3},$$ (54)
    $$Re=D_{M}U_{M}/\upsilon ,$$ (55)
    $$Ro=\frac{f_{M}D_{M}^{2}}{\upsilon }.$$ (56)
3. *Flow Measurement:*
  - *Reference Conditions:* (see p. 522)
    $${\dot{V}}_{M0}=\frac{f_{M0}}{{\bar{K}}_{M0}}=\frac{C_{1}D_{M0}^{3}f_{M0}}{\bar{St}}.$$ (59)
  - *Non-Reference Conditions:* (see p. 523)
    $${\dot{V}}_{M}=\frac{f_{M}}{{\bar{K}}_{M}}=\frac{C_{1}f_{M}D_{M}^{3}}{\bar{St}},$$ (60)
    $${\dot{V}}_{M0}=\frac{C_{1}f_{M}D_{M0}^{3}}{St}\left(\frac{\mu_{M0}}{\mu_{M}}\right)\frac{[1+2\alpha_{M}(T_{M}-T_{M0})]\left[1+\frac{(P_{M}-P_{M0})D_{M0}}{t_{M0}E_{M}}\right]}{[1+3\alpha_{F}(T_{M}-T_{M0})]\left[1-\frac{\left(P_{M}-P_{M0}\right)}{E_{F}}\right]}.$$ (65)
